# Patient Clothing as a Healing Environment: A Qualitative Interview Study

**DOI:** 10.3390/ijerph18105357

**Published:** 2021-05-18

**Authors:** Seonju Kam, Youngsun Yoo

**Affiliations:** Department of Clothing and Textiles, Kyung Hee University, Seoul 02447, Korea; kamjoo@empas.com

**Keywords:** hospitals, patients, clothing

## Abstract

Patients’ emotional responses to the hospital environment can be considered as important as medical technology and equipment. Therefore, this study investigated their experiences to determine whether the pattern using hospital identity (HI) elements, a widely used design method for patient clothing in university hospitals, can affect their emotional response and contribute to healing. It aimed to identify whether controlling the motif characteristics, arrangement, and spacing in this pattern design, and the direction between motifs, could be a method to design patient clothing for healing. To investigate patients’ emotional response and suggestions for patient clothing design, an interview-based qualitative approach was used. In-depth interviews were conducted with 12 patients discharged from Kyung Hee University Hospital Medical Center (KHUMC), Seoul. The interview questions consisted of two parts. One part featured questions about participants’ emotional responses to the medical environment and their latest patient clothing experience, and the other featured questions about their emotional response to, and suggestions for, the healing expression of pattern design using HI. The results confirmed that the motif characteristics, arrangement, and spacing, and the direction between motifs, influenced patients’ positive emotions and contributed to the healing effect. Therefore, when the HI elements of a medical institution are applied in the design of patient clothing with the characteristics of a healing design, patients perceive this as providing stability and comfort. The design of patient clothing becomes a medium that not only builds the brand image of medical institutions, but also enhances the quality of medical services centered on patient healing.

## 1. Introduction

The current medical environment is rapidly changing with the development of advanced technology, but provider-oriented medical services are still causing unsatisfactory results for patients. Patient satisfaction is evaluated as an important factor in the quality of medical services [1,2,3,4]. The interior design of a medical institution, including its furniture, ornaments, lighting, sound, color, landscape, and physical environment, including factors such as patient clothing and the uniforms of the medical personnel, are important factors in creating a healing atmosphere by stimulating the senses of patients and staff and creating memorable experiences [5].

As a physical environmental factor, patient clothing facilitates treatment and helps the healing of both mind and body [6]. Patient clothing is part of the medical environment and influences patients’ recovery. It also has a key role in building a rapport between patients and medical service [7]. Medical services are provided under conditions in which the patient and their family are physically and emotionally vulnerable, a fact that should be emphasized [8]. Healing requires the patient to recover physically and mentally, and can be approached using various characteristics related to human emotions, such as nature-friendliness, familiarity, stability, aesthetics, and relaxation [9]. A healing design for patient clothing can improve comfort and help patients to adapt to the treatment environment by considering their sensibility and spirit. Patient clothing with a healing design can alleviate stress, soothe negative emotions, and even play a role in helping the patient’s recovery by eliciting comfort, pleasure, and psychological and physiological stability.

Hospital identity (HI) is reflected in the hospital’s logo, symbol, signature, and slogan, and represents the visual image of the organization. Patient clothing reflecting HI functions to represent the hospital’s unique identity and differentiated brand image.

As existing patient clothing is mainly manufactured according to the HI plan, the hospitals’ position has usually received more attention than considerations regarding patient healing. Thus, there are many cases in which the patient’s psychological situation is not considered. This study aimed to identify a patient clothing design method that helps patients heal by modulating the motif of the pattern design associated with the HI factor. It investigated, through interviews, whether controlling the motif characteristics, arrangement, and spacing, and the direction between motifs in pattern designs using HI elements could be a method of designing patient clothing for healing.

## 2. Literature Review

### 2.1. Patient Clothing

Patient clothing originated in the 19th century when hospitals needed to improve hygiene and arrange suitable clothing for poor patients [10]. Patient clothing is the most important factor in maintaining a patient’s dignity and well-being and is one of the first factors encountered during hospitalization [11]. In addition, patient clothing is a part of the treatment environment, along with medical technology and equipment, and wearing patient clothing becomes an active therapeutic medium in the relationship between medical staff and patients by recognizing it as an object in the treatment process [7].

**Figure 1:**
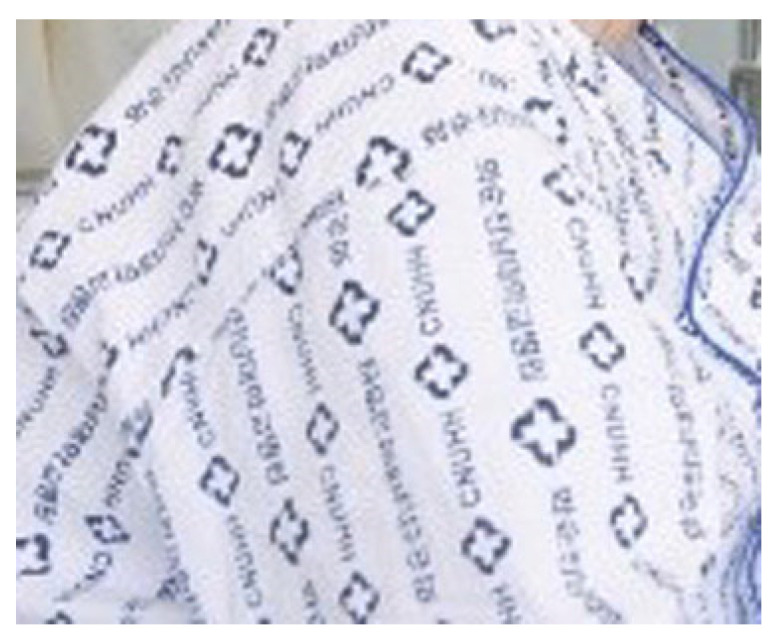
Striped pattern for patient clothing.

**Figure 2:**
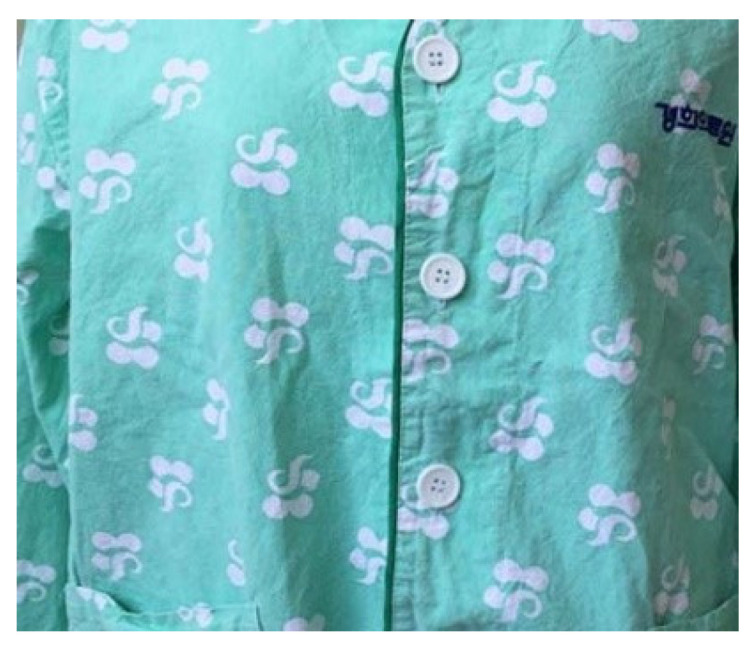
Block repeat pattern for patient clothing.

A number of earlier studies have mentioned the uncomfortable emotions that patients feel when wearing patient clothing. These have stated that the worn-out appearance of patient clothing affects the patient’s self-esteem [8] and causes discomfort by unintendedly exposing body parts [12]. Unnecessary exposure of the patient’s body to medical staff leads to a decrease in relative status, and patients feel shame because their privacy is not guaranteed [13]. Patients experience reduced opportunities for self-expression and feel depersonalized when wearing uniformly-shaped patient clothing [14]. Khorshid et al. [6] reported that when patients suffered stress and physical constraints during hospitalization and the recovery phase, positive emotions about clothing were effective in improving their healing and self-esteem. Therefore, when considered in terms of the treatment process, patient clothing is required not only to have a physical function for convenience of treatment, but also to influence the patient’s emotions by the quality of its design. Hospitals with a corporate system nowadays differentiate their brand value from their competitors by expressing the hospital’s identity in the design of patient clothing. The design of hospital patient clothing in Korea reflects brand identity and image by utilizing logos and symbolic marks. Figure 1 shows hospital clothing designed with the hospital’s logo and mark in a striped pattern, and Figure 2 shows clothing designed with a block repeat pattern. This was composed of an ordered image in which signatures and logos of the same size were arranged in repeating patterns. This seems to be related to the intention of most hospitals to promote favorable images such as trust, quality, and reputation. However, even if patient clothing reflecting HI can help differentiate hospitals at the point of contact with the customer, considering that the final goal of the relationship between the hospital and the patient is the patient’s healing, a design plan that can offer physical and psychological comfort should be developed.

When designing patient clothing, an emotional approach is possible through the visual image of the clothing and its design elements [15]. In addition, a style that changes the angle or arrangement of logos or logos related to the hospital’s visual identity can increase the awareness of movement and promote visual engagement by customers [15]. Moreover, the motif’s line, shape, and size, and the spacing between the motif and the background determine the feeling when looking at the pattern [16]. In other words, it is possible to provide a quality service with a design that considers the patient’s sensibilities and psychological state by changing the angle or interval of the pattern repeats while maintaining the same core design elements of HI. Patient clothing design can be promoted as a service that improves the psychological well-being of the patient while adding quality through design that considers the patient’s sensibilities and dignity as well as the basic function of ease of treatment.

### 2.2. Healing Design Approach

Healing is a lively process that involves regaining physical and mental integrity, recovery, and rehabilitation. Healing takes place at several levels of the human system, including the mental, physical, emotional, and spiritual [17]. The care environment has the potential to reinforce an individual’s inner strength, and a visually comfortable care environment has a positive effect on recovery after surgery [8,18]. The concept of healing design as a means of treatment is related to solving the patient’s psychological and physical needs and improving the patient satisfaction [19]. The environment has the potential to reinforce an individual’s inner strength and aid in healing by facilitating or enhancing the patient’s behavior [17,20]. Many previous studies have mentioned that it is helpful to introduce a healing environment into physical design.

Healing design includes many aspects that positively influence people to achieve healing goals. It is mainly used in medical facilities, such as hospitals, sanatoriums, healing spas, and retiree homes, for a positive impact on mental and physical health. In the design of patient clothing, research is needed to consider the interconnection between the physical and mental factors that prioritize healing.

On investigating the literature on design approaches for healing roles, Harris et al. [5] reported that the physical environment has an important role in the hospital experience and causes hospitals to function as healing spaces, thus influencing patient satisfaction. In terms of interior design, comfortable and functional equipment and furniture provide home-like comfort, and aesthetically pleasing decoration, art, and a spacious layout to accommodate visitors were factors that increased the patient’s satisfaction with the hospital. In addition, exposure to nature is said to combat mental fatigue and aid healing.

Timmermann et al. [21] found that positive sensory impressions in the hospital environment significantly affected mood and were largely obtained from maintaining patient identity and positive thoughts and emotions. In addition, they found that from the viewpoint of healing, natural and aesthetic decoration helps to maintain the patient’s identity, and that patients who can see pleasant, scenic views of nature through their hospital window develop positive thoughts and emotions.

Schreuder et al. [22] mentioned spatial comfort, privacy, and safety among the important design elements for a healing environment. The spatial comfort of the patient relates to the personalization of space, an aesthetic interior design, and the access to nature; moreover, privacy, and safety affect the patient’s well-being. Riisbøl et al. [23] studied a design method that provides healing architecture for patients, their relatives, and nurses. Sensory impressions are induced in patients through the atmosphere of the visit, the view of natural surroundings, and the provision of privacy. The aesthetics experienced in the wall decoration and the room color, as well as the atmosphere, influenced the experience of well-being and the quality of treatment; the resulting comfort gave patients a home-like familiarity and reduced the stress generated in the clinical hospital environment. Privacy is related to space, and the healing effect may improve on separating the space with a partition or curtain between patients.

**Figure 3:**
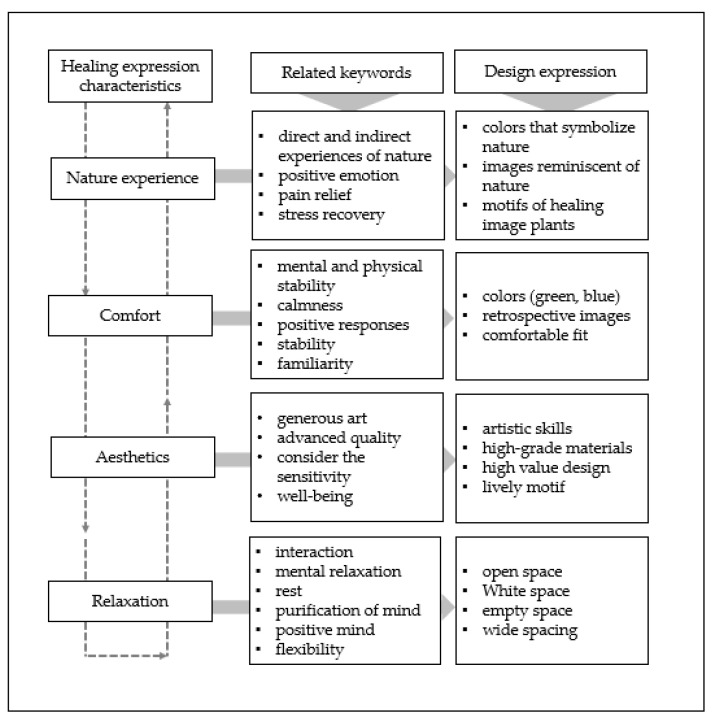
Summary of the design expression characteristics that influence healing through literature review studies.

Based on these studies, we explored theories related to “nature experience,” “comfort,” “aesthetics,” and “relaxation” as elements for healing design. Figure 3 shows the design expression characteristics and the design methods that influence healing, which we identified through a review of the literature on the following four characteristics.

#### 2.2.1. Nature Experience

Contact with nature can benefit personal health, and patients tend to experience recovery by looking at the natural environment [18,24,25,26]. For patients, experiences of nature can influence healing by reducing anxiety, anger, or negative emotions and inducing positive emotions. Numerous previous investigations have shown that exposing patients to nature has a positive effect on pain relief and healing, and that a brief look at nature can lead to a quick and meaningful recovery from stress [27]. In a study of patients recovering from appendectomy by Park and Mattson [28], patients in hospital rooms with plants and flowers had a significantly reduced intake of analgesics after surgery than those in hospital rooms without plants and flowers. Blood pressure, heart rate, pain, anxiety, and fatigue were lowered, and positive feelings and satisfaction with the hospital room were higher. Ulrich et al. [9] confirmed that colors symbolizing nature and images reminiscent of nature sustain or increase positive emotions such as comfort and calm and reduce negative emotions that cause worry and stress. Totaforti [29] stated that plants (especially roses), natural ventilation, natural light, and environmental design that can contact nature improves the work efficiency and organizational ability of hospital workers as well as the well-being of patients. Cliff Goldman and Louise Russell attempted to combine textile design with a healing environment, and developed a “healing fabric” arranged in a repeating pattern using life-size images of healing plants such as eucalyptus, silver dollar plant, bamboo, and jasmine [30].

#### 2.2.2. Comfort

Patients are likely to experience anxiety and feel vulnerable during hospital stays due to the unfamiliar sensations associated with the medical environment, which is mainly white color [23]. In addition, lack of visual or auditory privacy can cause discomfort [22]. In particular, patient clothes are often designed for medical treatment purposes and are in the form of pajamas or a gown for ease of treatment, which can cause anxiety about exposure if worn without underwear. For the safety of patients, patient clothing design must proper ease of access to enable comfortable treatment, but must also create a psychological environment that allows the patient to feel cared for and provides emotional comfort [6]. Color is an important design element and has a strong relationship with emotions [31]. Green, reminiscent of plants, and blue, reminiscent of the sky and water, are quiet and positive, and they encourage stability [32]. Emotional stability can be achieved by providing familiarity and comfortable environmental design. A study on the design of nursing homes found that elderly people preferred a retro-style flower design, which was able to bring memories of home and offered familiarity and comfort [33]. The design of patient clothing requires the development of patterns and the use of colors that can enhance psychological comfort, along with clothing design that avoids physical discomfort.

#### 2.2.3. Aesthetics

An attractive environment has the ability to distract attention and help patients recover from mental fatigue. The patient’s satisfaction with the hospital increases when the space they are in is aesthetically pleasing and comfortable [34]. An art-rich environment can be seen as therapeutic, providing a means to alleviate physical discomfort, emotional pain, and mental crises. Using art as a healing tool can improve the outcome and quality of treatment, and art plays an important role in rapid recovery [25]. The design of the elements comprising the physical environment that result in sensory stimulation, such as buildings, equipment, furniture, signboards, colors, art, landscapes, and clothing, is perceived to indicate a hospital’s quality of care and can positively influence patient healing [35]. Since patients experience the patient clothing design directly, the aesthetics that can inspire positive emotions in them, such as images representing optimism, vitality, and humor, should be considered to improve the healing system [36]. Feodoroff, a designer who developed a functional patient suit “Original Healing Threads” for women with cancer, emphasized the importance of design considering the aesthetic sensitivity of patients by commenting that when you feel like you look good, you will get better [37]. For patient clothing designs, aesthetics should be addressed as a different concept from that of general fashion. Above all, patient clothing should be designed to help heal by adjusting design elements by reflecting the emotions and tastes of patients.

#### 2.2.4. Relaxation

Lau et al. found that viewing an open space would clear the minds of users who want to relax, arouse positive attitudes, and relieve tense nerves [38]. The white space in advertising design leads users to interact with the design in a relaxed emotional state, increasing their favorable perceptions about product quality and reliability [39]. Relaxation is not only related to the comfortable fit of the patient clothing design, but also to the healing effect since it allows the patient to experience the emotion of comfort in the white space constituting the pattern design.

## 3. Methodology

### 3.1. Design and Interview Participants

The study was designed to explore user suggestions for patient clothing design using a qualitative approach that involved conducting individual in-depth interviews. Before recruiting participants, approval was obtained from the administrative department of KHUMC, Seoul. The target group of study participants was cured patients of KHUMC who had completed the discharge procedure. Potential participants and their families were informed about the purpose of the study and the interview method to be used. Subsequently, 12 participants voluntarily agreed to participate in the study. Their interviews were conducted from 1–30 December 2020.

### 3.2. Procedure

The stimulus was based on the patient clothing currently used by KHUMC (Table 1), which was designed by this research team with the support of the KHUMC Fund. To examine whether the current patient clothing pattern design using HI elements embodies the characteristics of the healing theory effectively, and to explore user opinions on what they would consider an improved healing design, the healing design was based on the shape and color of the current patient clothing. Six stimuli related to four characteristics were added.

Semi-structured questions were used in the interviews. Each interview lasted about 30–40 min. The interview questions consisted of two parts. One part had open-ended questions to explore the participants’ emotional response to the medical environment and to the current patient clothing experience. The other part had questions on the participant’s emotional response to, personal preference for, and suggestions regarding the healing expression of pattern design using HI; the stimulus was used along with the questions. The two-part questions were built around the keywords related to design expression characteristics that influence healing shown in Figure 3.

**Figure 4:**
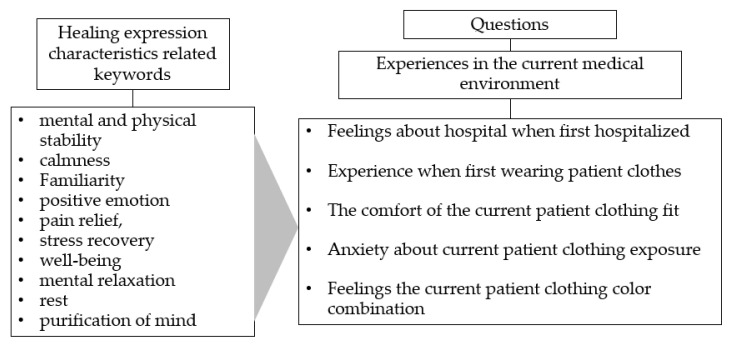
Questions used to elicit patients’ feeling about the current medical environment.

Figure 4 summarizes the questions used to elicit patients’ feelings about the current medical environment and their clothing experience. The keywords in Figure 4 were alternately referred to as additional questions to facilitate the participant’s answers during the interview process and to more accurately identify the emotions associated with healing characteristics.

The interview about the users’ emotional response, personal preference, and suggestions for the healing characteristics of pattern design using HI were conducted by presenting a stimulus. During each interview, a tablet, which had a photograph of the current patient clothing, and a printed photograph of the patient clothing with manipulated patterns to reflect various healing characteristics, were used.

**Figure 5:**
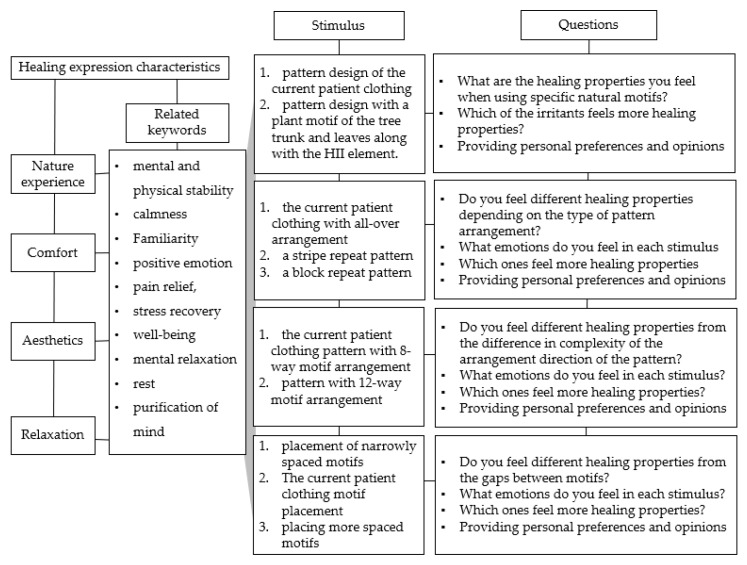
The question-extraction process for the patient’s emotional response to pattern design using HI.

Figure 5 shows the process used to elicit answers from the interviewees regarding their emotional response to pattern design using HI. The following are the four design directions used as a stimulus. Questions about plant motifs relate to the nature experience design direction. This design direction was based on the findings in the literature that images reminiscent of nature maintain or increase positive emotions and influence healing [9]. An image using only the pattern representing the hospital’s brand identity was used, and tree trunks and leaves were added to create a stimulus that realistically expresses the nature image.

Regarding the second research direction, comfort, studies have shown that a familiar and comfortable design improves emotional stability [23]. While the all-over arrangement of patterned motifs is related to comfort, directional arrangements in which the motifs are repeatedly arranged along the length of the garment or in parallel have significant and influential psychological effects [15]. Additional stimuli were created with a striped repeat pattern and a block repeat pattern for comparison with the current patient clothing.

The third design direction is aesthetics. Designs that aesthetically apply humor or vitality can improve healing by creating positive emotions in the patient [36]. In pattern design, the angle at which a motif is placed can affect the patient’s psychological vitality. We added the stimulus of the all-over pattern placed at 12 angles to compare its effect with those of the current patient clothing in the pattern placed at 8 angles.

The fourth design direction is relaxation. The white space in a design stimulates patients’ sense of leisure, helping to calm the mind. Designs with wide margins around the motif can lead to a positive evaluation and image improvement [38]. To compare the differences between narrowly spaced pattern motifs and wider spaced motifs, we added three images with different margins, as stimuli.

## 4. Result

A total of 12 adults participated in the interviews, among whom 10 were working age, and two were aged 65 years or above. The male to female ratio was 2:10. Interviewees were assigned alphabets as initials to ensure anonymity.

### 4.1. Experiences and Opinions in the Medical Environment

A number of prior studies have emphasized the healing environment by mentioning patients’ hopes for healing when hospitalized and their anxiety about an unfamiliar environment. In this study, most patients felt relief, expectation, and hope for healing as soon as they were hospitalized, and at the same time, there was fear or discomfort in the unfamiliar environment of the hospital. Most interview participants mentioned their hopes for healing and positive feelings.

“I’m relieved that the medical staff is taking care of me closely, but I’m nervous about the treatment. The treatment process could be painful, and it was unfamiliar because it wasn’t home. But seeing the tidy room made me feel stable…”

(Interview Participant D)

Interview participants seemed to be trying to accept their anxiety about their disease and unfamiliar and uncomfortable feelings about the new environment with trust in the treatment staff, and they felt a sense of stability in a ward arranged as a healing environment.

In response to the question about their emotions when they first put on the patient clothing, most of the participants answered that they felt comfortable, clean, and comfortable to work. In contrast, one respondent answered that they were worried about the deprivation of their social status, while another worried about exposure when wearing patient clothes, but most of the interviewees mentioned the feeling of being cared for.

“The patient clothing was comfortable, clean, and pleasant, but it looked cheap.”

(Interview Participant A)

“When I wear patient clothing, I felt a little deprived of my self-esteem because my job doesn’t appear anyway. But I felt more cared for.”

(Interview Participant K)

Most of the interview participants commented that the first feeling associated with wearing the patient clothing was that of being treated and that it was comfortable, and a clean and pleasant feeling was mentioned next. They were provided with fresh patient clothes on a regular basis and they could be replaced at any time in case of contamination.

Studies have identified that the problems with using conventional patient clothing are that it induces a feeling that their social status has reduced, anxiety about physical exposure, and negative emotions, such as shame, in patients. This was associated with negative feelings that privacy was not guaranteed. Most of the 12 interview participants were satisfied with the shape and structure of the patient clothing. However, there were a few interview participants who mentioned size-related discomfort, which was related to the length of the sleeves and pants.

“When wearing patient clothing, it’s comfortable, but the ankles are exposed because the sleeves are short and the pants are short.”

(Interview Participant I)

In terms of shape, interview participants expressed satisfaction with the current clothing regarding their concerns about exposure and comfort, in particular for arm activity. This seems to have been improved by taking into account patient dissatisfaction through theoretical research.

In theoretical research, color is an important aspect of patient clothing because it can access the emotions, and certain colors influence common emotions. Most of the participants expressed the opinion that they felt clean and hygienic in clothing with a white background color. Some of the participants talked about the fact that any contamination was easily visible because of the white background, but this was different from the negative emotion provoked as a result of the contamination.

“Because the background color is white, contamination is easy to see. If there is still contamination, then you can replace it and wear clean clothes immediately.”

(Interview Participant C)

Most of the interview participants expressed positive opinions that the background color was white, and none of the participants mentioned that the clothes looked worn because of the color. As mentioned in previous studies, there were also opinions mentioning that white clothing looks cold. The blue and green colors used in the pattern motif are colors brought from the character mark representing the hospital’s brand identity, and are reminiscent of natural images. During the interview, the most common opinions about these colors were about the clean and fresh feeling they were associated with.

“It’s clean and fresh. That’s why it seems to be the color used a lot in patient clothing.”

(Interview Participant I)

Interviewees K, J, and F mentioned emotions they felt in response to the colors.

“The colors of the pattern feel calm and gentle, and there are positive and hopeful feelings. It feels unfamiliar, but it also feels warm. The color saturation feels refined and luxurious.”

(Interview Participant K)

There were some participants who mentioned aesthetics as an expression of refinement and luxury as an expression of aesthetics.

“The color scheme is fresh.”

(Interview Participant J)

“I think the color scheme is tacky.”

(Interview Participant F)

Various opinions were expressed regarding the color scheme, but colors are most often encountered by the general public, and it seems that positive opinions were expressed regarding personal preferences and experiences. The color of the current patient clothing is the color of the natural images among the HI’s character mark colors and aims to contribute to a healing effect.

### 4.2. Opinions of the Healing Characteristics of the Hospital Identity Motif Design

#### 4.2.1. Nature Experience

In theoretical research, the pattern has an effect of reminiscent of images and has a psychological influence based on the images in the pattern. Images reminiscent of nature sustain or increase positive emotions, which has a healing effect. Stimulus A, using only the pattern extracted from HI, and Stimulus B, with the addition of tree trunks and leaves, were presented as realistically expressing a nature image (Table 2). Interview Participant F, who commented on the nature environment featured in the pattern, said that seeing the floral design made them feel alive. Interview Participant B said that he chose Stimulus A because he felt good when he saw both stimuli and the nature image.

“I feel that Stimulus A is more stable. When I see the nature image in Stimulus B, it feels good, but it feels a bit distracting. It’s nice to have a lively feeling, but I think that the patient clothing is stable.”

(Interview Participant B)

Interview participants preferred Stimulus A. Among the reasons for choosing Stimulus A, they stated that they felt familiarity, comfort, neatness, and stability when looking at the pattern.

“I think a neat and stable feeling makes me more comfortable.”

(Interview Participant K)

“Stimulus B has the shape of a tree trunk and leaves, so it looks lively, but it looks a little messy and distracts, so my eyes are tired.”

(Interview Participant F)

There were more reasons expressed by the interview participants to feel positive emotions and a familiar, stable, and comfortable feeling after seeing the nature image, and in some cases, the nature image gave them a lively and energetic feeling. The nature image motif was not affected by whether the form representing natural objects was realistic or metaphorical, but it positively influenced the interview participants.

Among the elements expressing HI, pattern design A using only the magnolia character and signature was better received than pattern design B, which added a natural object motif unrelated to HI. We presume that if used properly, this may influence the healing effect of the clothing. As described above, the nature experience design direction may have a healing effect by positively affecting patients’ emotions. However, the introduction of excessive motifs hindered their emotional stability, and they perceived the image as cluttered. KHUMC’s HI used magnolia graphics. Thus, if the HI has images of natural objects, then designing patient clothing using HI will yield successful results.

#### 4.2.2. Comfort

In a theoretical study, a sense of being cared for comfortably and safely was positive for the patient [40]. Table 3 shows stimuli related to the arrangement of the pattern motifs. When interview participants talked about their preferences for the arrangement of the pattern motifs, the most important factor was comfort and stability. Most interview participants preferred the all-over arrangement, and none preferred the striped pattern often used in existing university hospitals.

“I like the all-over arrangement. It looks good and gives a sense of stability and comfort.”

(Interview Participant C)

The participant who preferred the block repeat arrangement said that the reason for his preference was that it felt stable and comfortable because the floral pattern was more recognizable than the block repeat arrangement.

“I like the friendly floral pattern in a block repeat arrangement, so it looks comfortable.”

(Interview Participant E)

In addition, the interview participants wanted a comfortable feeling, but tried to feel liveliness or dynamism with comfort rather than a rigid or stagnant feeling. They said that they felt stable and comfortable when they saw the all-over arrangement.

“The grid pattern also looks comfortable, but I like the current patient clothes with the all-over arrangement because they don’t feel stagnant. They feel lively.”

(Interview Participant L)

There was also a negative opinion of striped arrangement, because it gave a feeling of rigidity.

“The stripe pattern arrangement is like prison garb. The block repeat arrangement is distracting and it makes my eyes tired. The arrangement in all directions feels stable and my eyes are comfortable.”

(Interview Participant B)

“It’s uncomfortable to arrange hard or complicated arrangements in patient clothing.”

(Interview Participant K)

Based on this, most interview participant felt stable and comfortable when wearing the all-over pattern arrangement, and the floral motif was related to this arrangement, so there was a preference for the block repeat arrangement.

In the case of motifs designed using HI expression elements, it is thought that healing properties can be exhibited by applying different pattern arrangement methods accordingly. In a previous study, as regards the arrangement method, the image of the rigid stripe and block arrangement was mentioned as a negative image [15]; however, some interview participants expressed the opinion that the flower motif was stable when used with the block arrangement. It is inferred that they felt this way because this arrangement induced in them a feeling of closeness with nature, and it was reflected as a healing characteristic.

#### 4.2.3. Aesthetics

In previous studies, attractive patient clothing designs have been shown to give patients confidence. We investigated whether attractive and vibrant patterns are related to healing. Stimulus A is the all-over pattern placed at 8 angles, and Stimulus B is the all-over pattern placed at 12 angles Interview (Table 4). participants said that although they prefer moderate liveliness, motifs oriented in too many different directions feel more confusing than lively.

“B has too many tilt angles, so it looks uneasy. I like A because A has a stable angle.”

(Interview Participant H).

Interview Participant J said that the angles are so different that the pattern seemed rather complicated. Interview Participant K said that it while it was good to be all-over of a directional pattern, but too many varied angles make them feel dizzy.

Interview participants felt positive emotions with the direction of movement when there was some stability in the pattern direction, but distractions from too many angles produced negative emotions. Vibrancy can be used as a healing property that enhances patients’ self-confidence, but if it is excessive, it can reduce patients’ psychological stability. In this part of the analysis, it was also confirmed that the excessive use of elements in the pattern design of patients’ clothing could hinder the healing effect. To suggest a more appropriate number of elements or a design method, a follow-up study that uses more different cases of stimulus needs to be conducted.

#### 4.2.4. Relaxation

In previous studies, it was reported that blank space in an environment provides an opportunity for the user’s emotions to intervene. In the design of clothes, it is possible to provide a relaxed feeling for patients by adjusting the spacing of patterns. Table 5 shows three images with different the spacing of patterns, as stimuli. The interview participants felt a sense of relaxation with the blank spaces in the clothing, and they felt frustrated and tense when the gap between the patterns was narrow. Half of the participants chose the Spacing B of the pattern motif they were currently wearing, and four participants chose Spacing C when the spacing of the pattern was wider. The reason they preferred the spacing of the motifs of the current patient clothing is comfort.

“If the spacing of the patterns is adequate, the viewer feels relieved. If it’s narrow, it’s frustrating.”

(Interview Participant K).

“It’s stable because the pattern spacing is adequate. If it is too wide, it feels too relaxed, and if it is dense, it is complicated.”

(Interview Participant L).

If the gap between the patterns was narrow, interview participants felt frustration and a sense of complexity, and if the gap was too wide, they felt a feeling of looseness.

The preference for a pattern design with wide spacing between the motifs was common, and this may be related to previous studies indicating that the emotions felt in response to the blank spaces may be different for each individual. In previous studies, the space itself felt empty, and luxury was recognized in the blank spaces.

## 5. Discussion

This study attempted to identify methods of designing patient clothing that can contribute to patient treatment based on questions about the pattern design related to HI elements, the motif characteristics, arrangement, and spacing, and the direction between motifs. Through in-depth interviews, it was found that in patient clothing design, design adjustments such as the use of the motif characteristics, arrangement, and spacing, and the direction between motifs, influence the healing effect. This focuses on the characteristics of healing sensibility, such as nature experience, comfort, aesthetics, and relaxation. Interestingly, in the case of patient clothing design, the patient’s emotional healing is an important factor, and appropriate adjustment of the size or quantity of the design motifs was required. First, the healing properties of nature experience can be employed by utilizing nature motifs such as flowers or leaves. We have confirmed that excessive use of many motifs with different characteristics hinders healing due to the complexity of the pattern. This finding is consistent with the theories that seeing nature induces positive emotions and reduces stress [9], but it contradicts the theory that predicts that the more immersed in environmental distraction that patients are, the greater is their pain reduction [41]. Second, the in-depth interviews revealed that the interview participants gained psychological stability through the all-over arrangement, which is consistent with the literature [15]. When using images of natural objects or HI expression elements, the all-round arrangement conveyed a sense of stability. The interview participants felt comfortable because the flower patterns in this arrangement gave them a sense of familiarity. This is connected with the fact that intimacy has an influence on comfortable emotions [33]. In addition, in the case of using a nature object image or using an HI expression element, the block repeat arrangement method can also provide stability via the nature property or arrangement. Third, a sense of stability is recognized when an appropriate number of angles are used in the design of the pattern direction. Various distracting angles create anxiety. The direction of the pattern expresses liveliness and can increase the confidence of patients by acting as a charming aspect of patient clothing design. Therefore, the direction of the pattern needs proper adjustment. This study showed that although visual distraction reduces both pain and anxiety stress [9], the degree of distraction requires appropriate adjustment and differs between the interview participants. Fourth, the middle-width spacing of the motifs was the most preferred among the three stimulus examples, followed by the widest space. Our results support the assertion that blank spaces allow for a sense of relaxation and are felt as luxuriousness or sophistication [42].

In a number of previous studies on patient clothing, shame due to exposure, poor clothing design, and reduced social status were mentioned as negative effects. These dissatisfaction factors were improved in the KHUMC patient clothing, but young patients were concerned about exposure as a result of white clothing. The use of white was requested by the medical provider to aid in hygiene management of patient clothing, but the fact that patient clothing should be user-centered needs to be considered when designing clothing in the future. Most of the participants felt positive about the patient clothing currently worn. This is thought to be because the design plan was made after referring to previous studies prior to the production of the patient clothing.

In this study, the perspectives of 12 people with experience in patient clothing were obtained through interview. This methodology may not be suitable for, or implemented in, other settings. However, we have shared an important finding, that designing patient clothing by analyzing their experiences can contribute to healing.

## 6. Conclusions

Most of earlier studies related to patient clothing have been conducted from the providers’ point of view, so our understanding of users’ requirements was insufficient. This study is meaningful in that it has gone through the process of confirming existing theory through in-depth interviews with patients using actual patient clothing. This study is significant in that it revealed that when designing patient clothing using HI elements, motif characteristics, arrangement, and spacing, and the direction between motifs, must be considered, since these positively affect patients’ healing. In the future, we will apply the results of this study to patient clothing and develop and thoroughly review the designs in more case studies.

## Figures and Tables

**Table 1:** Current patient clothing.

Textile Design	Outfit
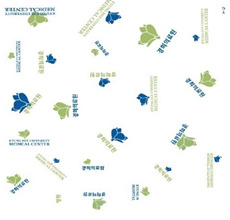	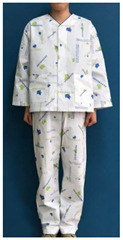
Healing expression characteristics: A character mark symbolizing a magnolia flower, which is an element of HI, was introduced as a motif, and among the colors constituting the character mark, blue and green, noted to be related to psychological stability, were adopted as the colors of healing. HI: The hospital’s character mark and signature were used as the motif for the pattern design. Protection of patient privacy: The neckline depth was 1.5 cm higher than that of existing patient clothing to reduce exposure during bowing the upper body. The existing patient clothing has a deep neckline for convenience when using medical devices such as stethoscopes. To reduce exposure from the gaps between the buttons, the overlapping part of the front fastening was made wider than that of the existing patient clothing. Activity and sustainability: To better consider the patient’s activity and comfort levels, the width between the shoulders was greater than that of the existing clothing and the height of the sleeves was lowered. This was taken from the flat pattern of Hanbok, and affects the order of sewing in mass production, improving economic efficiency and sustainability. Hygiene management: A white background was chosen to easily deal with contamination.	

**Table 2:** Nature experience healing characteristics: the patient clothing design stimulus.

A		B	
Current patient clothing: Magnolia character mark and signature used in HI		The magnolia character mark, signature and motifs in HI. Tree trunks and leaves symbolizing life form the pattern	
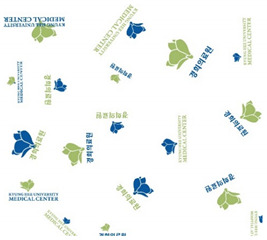	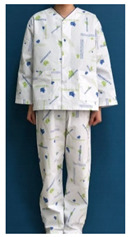	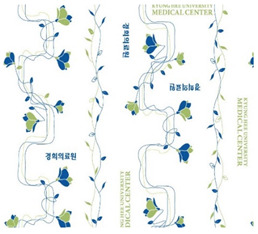	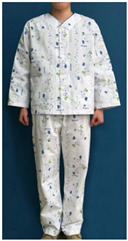

**Table 3:** Comfort healing characteristics: patient clothing design stimulus.

A	B	C
Current patient clothing: All-over arrangement	Stripe arrangement	Block repeat arrangement
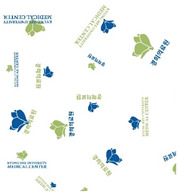	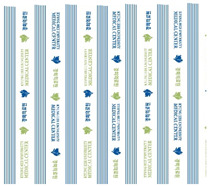	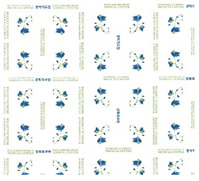
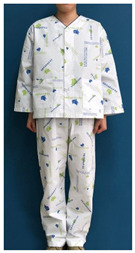	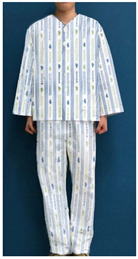	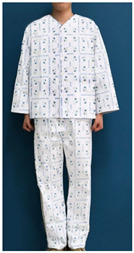

**Table 4:** Aesthetic healing characteristics: Patient clothing design stimulus.

A		B	
The current patient clothing pattern motif		More various angular directions than current patient clothing	
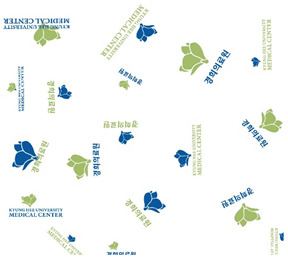	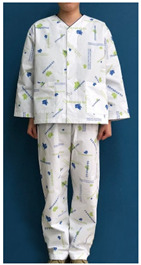	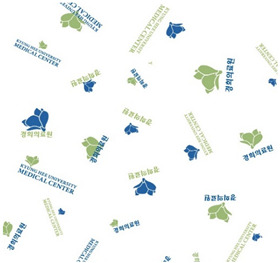	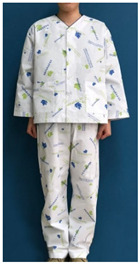

**Table 5:** Relaxation healing characteristics: patient clothing design stimulus.

A	B	C
Narrow gap between pattern motifs	Current patient clothing pattern motifs	Wide space between pattern motifs
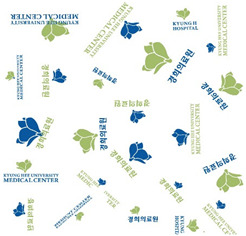	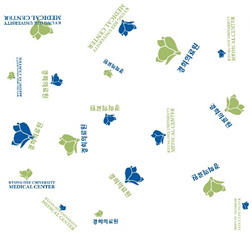	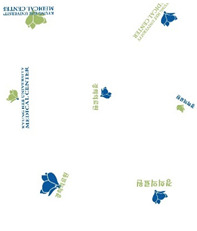
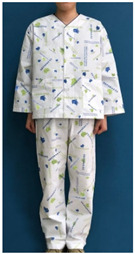	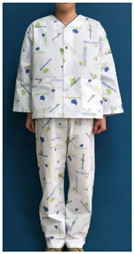	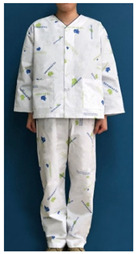

## Data Availability

Data available in a publicly accessible repository.

## References

[1] Marimon F., Gil-Doménech D., Bastida R. (2019). Fulfilment of expectations mediating quality and satisfaction: The case of hospital service. Total Qual. Manag. Bus. Excell..

[2] Moliner M.A. (2009). Loyalty, perceived value and relationship quality in healthcare services. J. Serv. Manag..

[3] Nekoei-Moghadam M., Amiresmaili M. (2011). Hospital services quality assessment. Int. J. Health Care Qual. Assur..

[4] Jennings S. (2005). What really matters to healthcare consumers. J. Nurs. Admin..

[5] Harris P.B., Ross C., McBride G., Curtis L. (2002). A place to heal: Environmental sources of satisfaction among hospital patients. J. Appl. Soc. Psychol..

[6] Vaskooi-Eshkevari K., Mirbazegh F., Soltani-Kermanshahi M., Sabzali-Poursarab-Saeedi M., Alipour S. (2019). Customized patient clothing and patient satisfaction. Int. J. Health Care Qual. Assur..

[7] Topo P., Iltanen-Tähkävuori S. (2010). Scripting patienthood with patient clothing. Soc. Sci. Med..

[8] Esposito A. (2017). Hospital Branding in Italy: A Pilot Study Based on the Case Method. Health Mark. Q..

[9] Ulrich R.S., Zimring C., Zhu X., DuBose J., Seo H.B., Choi Y.S., Quan X., Joseph A. (2008). A review of the research literature on evidence-based healthcare design. HERD.

[10] Iltanen S., Topo P. Ethical implications of design practices. The case of industrially manufactured patient clothing in Finland. Proceedings of the Konstfack of the Conference.

[11] Rabin J.M., Farner K.C., Brody A.H., Peyser A., Kline M. (2019). Compassionate coverage: A patient access linen system. J. Patient Exp..

[12] Karro J., Dent A.W., Farish S. (2005). Patient perceptions of privacy infringements in an emergency department. Emerg. Med. Australas..

[13] Nayeri N.D., Aghajani M. (2010). Patients’ privacy and satisfaction in the emergency department: A descriptive analytical study. Nurs. Ethics.

[14] Edvardsson D. (2009). Balancing between being a person and being a patient—A qualitative study of wearing patient clothing. Int. J. Nurs. Stud..

[15] Davis M. (1987). Visual Design in Dress.

[16] Kim S.M., Jeong S.J. (2008). A Study of the Changes in Dress Wearers’ Images in Relationto the Changes in the Size and Area Ratio of Polka Dots Relative to Coloration. J. Korean Soc. Costume.

[17] Jonas W.B., Chez R.A., Duffy B., Strand D. (2003). Investigating the impact of optimal healing environments. Altern. Ther. Health Med..

[18] Ulrich R.S. (1984). View through a window may influence recovery from surgery. Science.

[19] Stichler J.F. (2008). Healing by design. J. Nurs. Admin..

[20] Leather P., Beale D., Santos A., Watts J., Lee L. (2016). Outcomes of environmental appraisal of different hospital waiting areas. Environ. Behav..

[21] Timmermann C., Uhrenfeldt L., Birkelund R. (2013). Cancer patients and positive sensory impressions in the hospital environment—A qualitative interview study. Eur. J. Cancer Care.

[22] Schreuder E., Lebesque L., Bottenheft C. (2016). Healing environments: What design factors really matter according to patients? An exploratory analysis. HERD.

[23] Riisbøl M.F., Timmermann C. (2020). User consultation and the design of healing architecture in a cardiology department—Ways to improve care for and well-being of patients and their relatives. Nord. J. Arts Cult. Health.

[24] Wilson E.O. (2017). Biophilia and the conservation ethic. Evolutionary Perspectives on Environmental Problems.

[25] Ulrich R.S., Gilpin L. (2003). Healing Arts. Putting Patients First.

[26] Kozarek R.A., Raltz S.L., Neal L., Wilbur P., Stewart S., Ragsdale J. (1997). Prospective trial using virtual vision as distraction technique in patients undergoing gastric laboratory procedures. Gastroenterol. Nurs..

[27] Malenbaum S., Keefe F.J., de Williams A.C.C., Ulrich R., Somers T.J. (2008). Pain in its environmental context: Implications for designing environments to enhance pain control. Pain.

[28] Park S.-H., Mattson R.H. (2008). Effects of flowering and foliage plants in hospital rooms on patients recovering from abdominal surgery. HortTechnology.

[29] Totaforti S. (2018). Applying the benefits of biophilic theory to hospital design. City Territ. Arch..

[30] Carroll R.A. (2005). Applying design and color to healing. Nurs. Homes.

[31] Güneş E., Olguntürk N. (2020). Color-emotion associations in interiors. Color Res. Appl..

[32] Elliot A.J., Maier M.A. (2016). Color and psychological functioning. Curr. Dir. Psychol. Sci..

[33] Stevens K., Fröis T., Masal S., Winder A., Bechtold T. (2016). Design and colour preferences for older individuals in residential care. Res. J. Text. Appar..

[34] Urlich R., Zimring C., Quan X., Joseph A., Choudhary R. (2004). The Role of the Physical Environment in the Hospital of the 21st Century: A Once-in-a-Lifetime Opportunity.

[35] Steinke C. (2015). Assessing the Physical Service Setting. HERD.

[36] Ingrid E., Pavla G., Martina M. (2018). Healing and therapeutic Landscape Design—Examples and experience of medical facilities. ArchNet-IJAR.

[37] Greenberg J. (2005). Spirited sisters heal with fashion. WWD.

[38] Lau S.S.Y., Gou Z., Liu Y. (2014). Healthy Campus by Open Space Design: Approaches and Guidelines. Front. Archit. Res..

[39] Douglas O.G., Pracejus J.W., O’Guinn T.C. (2012). Print Advertising: White Space. J. Bus. Res..

[40] Campbell D.E. (1979). Interior Office Design and Visitor Response. J. Appl. Psychol..

[41] McCaul K.D., Malott J.M. (1984). Distraction and Coping with Pain. Psychol. Bull..

[42] Sharma N., Varki S. (2018). Active White Space (AWS) in Logo Designs: Effects on Logo Evaluations and Brand Communication. J. Advert..

